# Hydrogen sulphide and mild hypothermia activate the CREB signaling pathway and prevent ischemia-reperfusion injury

**DOI:** 10.1186/s12871-015-0097-6

**Published:** 2015-08-18

**Authors:** Hai-bin Dai, Xiangjun Ji, Si-hai Zhu, Yi-min Hu, Li-dong Zhang, Xiao-lei Miao, Ru-Meng Ma, Man-lin Duan, Wei-yan Li

**Affiliations:** 1 Department of Anesthesiology, Jinling Hospital, School of Medicine, Nanjing University, No. 305 East Zhongshan Road, Jiangsu Nanjing, 210002 China; 2 Department of Neurosurgery, Jinling Hospital, School of Medicine, Nanjing University, No. 305 East Zhongshan Road, Jiangsu Nanjing, 210002 China; 3 Department of Experimental Surgery, Tangdu Hospital, Fourth Military Medical University, Xi’an, China

**Keywords:** Hydrogen sulphide, Hypothermia, N-methyl-D-aspartate receptor, Cyclic AMP response element binding protein, Brain ischemia, Reperfusion injury

## Abstract

**Background:**

Both hydrogen sulphide (H_2_S) and mild hypothermia have been reported to prevent brain damage caused by reperfusion assault through regulating the N-methyl-D-aspartate receptor (NMDAR). However, the relationship between the two treatments and how they exert neuro-protective effects through NMDARs remain to be elucidated.

**Methods:**

Transient cerebral ischemia was induced using the Pulsinelli four-vessel occlusion method. We used sodium hydrosulphide (NaHS) as the H_2_S donor. We randomly divided 100 Sprague–Dawley rats into five groups of 20: Sham operation group (Sh), normothermic (36-37 °C) ischemia group (NT), mild hypothermic (32-33 °C) ischemia group (mHT), normothermic ischemia combined with NaHS treatment group (NT + NaHS), and mild hypothermic ischemia combined with NaHS treatment group (mHT + NaHS). After 6 hrs of reperfusion, rats were decapitated and hippocampus samples were immediately collected. We measured NR2A (GluN1), NR2B (GluN2) and p-CREB protein levels using western blotting. We further analyzed BDNF mRNA expression by real-time PCR. Hematoxylin and eosin (HE) staining was used to examine pyramidal cell histology at the CA1 region. All statistical analyses were carried out by ANOVA and LSD *t*-test as implemented by the SPSS 13.0 software.

**Results:**

In the four test groups with ischemia-reperfusion, hippocampal H_2_S concentration increased following treatment, and administration of NaHS further increased H_2_S levels. Moreover, administration of both NaHS and mild hypothermia resulted in up-regulation of NR2A and NR2B protein expressions, as well as p-CREB protein and BDNF mRNA levels. At the cellular level, NaHS and mild hypothermia groups exhibited lower damage caused by ischemia-reperfusion in the CA1 region of the hippocampus. The strongest protective effect was observed in rats treated with combined NaHS and mild hypothermia, suggesting their effects were additive.

**Conclusion:**

Our results support previous findings that hydrogen sulphide and mild hypothermia can prevent ischemia-reperfusion injury. Both treatments caused an up-regulation of NMDA receptors, as well as an elevation in p-CREB protein and BDNF mRNA levels. Thus, hydrogen sulphide and mild hypothermia may provide neuro-protective effect through activating the pro-survival CREB signaling pathway.

## Background

Ischemia-reperfusion injury occurs when blood supply returns following a period of ischemia. Reperfusion can damage tissues and cells through a series of reactions that lead to inflammation and oxidative damage [1]. *N*-methyl-D-aspartate (NMDA) receptor-mediated excitotoxicity has been shown to be a critical factor in neuronal damage following ischemia-reperfusion insults such as stroke or brain trauma [2]. The NMDA receptor is a plasma membrane channel that exists as a heteromeric complex composed of two glycine-binding NR1 subunits in combination with various glutamate binding NR2 subunits (A-D). These NR2 subunits vary according to the brain region and confer distinct electrophysiological and pharmacological properties on the receptors. NR2A (GluN1) and NR2B (GluN2) are predominant in the adult forebrain- the region where stroke most frequently occurs- and have opposing roles in influencing the direction of synaptic plasticity [3]. NR2A is predominantly located at synapses, whereas NR2B is mainly found at extrasynaptic locations. Evidence from mature cortical neuron cultures and rat models of focal ischemic stroke indicated that activation of NR2A-containing NMDA receptors promoted neuronal survival, while activation of receptors containing NR2B promoted neuronal apoptosis due to excitotoxicity [3]. Thus, the activation of NMDA-receptors at synaptic locations likely promotes neuronal survival, whereas activation of NMDARs at extrasynaptic locations may promote neuronal death. Synaptic NMDARs are associated with the CREB and PI3K-Akt signaling pathways, which may mediate their pro-survival effects [4]. Conversely, the pro-apoptotic pathways associated with extrasynaptic NMDARs appear to function predominantly through inhibition of CREB or activation of nNOS or JUN [5–7].

Hydrogen sulphide is an endogenous signaling gas molecule that has been shown to regulate functions of cardiovascular and central nervous systems [8, 9]. Moreover, it is becoming apparent that regulation of H_2_S can have cytoprotective effects in models of reperfusion injury. Indeed, it has been suggested that H_2_S can selectively regulate the function of NMDARs [10, 11]. It has also been shown to protect against traumatic brain injury and cerebrovascular disease under conditions of mild hypothermia at 30 ~ 33 °C. Thus, modulating H_2_S or NMDARs may have significant therapeutic potential. However, the relationship between H_2_S and hypothermia and how they exert neuro-protective effects through NMDARs remain to be clarified.

In this study, we utilized a global cerebral ischemia-reperfusion model of rat hippocampus to investigate the underlying mechanisms of neuro-resuscitation provided by H_2_S and hypothermia treatments. We showed that following global cerebral ischemia-reperfusion, both exogenous administration of the H_2_S donor compound, NaHS, and mild hypothermia prevented cellular damage. We also showed that H_2_S and mild hypothermia treatments induced up-regulation of downstream effectors of the CREB pathway, including p-CREB and BDNF, suggesting these treatments may activate the CREB pathway to exert their effects.

## Methods

### Experimental animals

We used 100 three month old male Sprague-Dawley rats (SD rats) weighing 250 ~ 300 g (Nanjing General Hospital of Comparative Medicine Branch) for all experiments. Rats were maintained at 22 ~ 25 °C; light-dark cycle 12 h/12 h; humidity 50 ~ 60 %; given free diet and were allowed to adapt to the environment for one week before the experiment. Rats were subjected to fasting and water deprivation for 12 h prior to the experiment. All experimental procedures were approved by the Ethics Review Committee of Jinling Hospital, Nanjing University and every effort was taken to minimize pain or discomfort of rats.

### Ischemia model and drugs

Four-vessel occlusion cerebral ischemia was induced as previously described [12]. Briefly, following anesthesia with chloral hydrate (300 mg/kg, i.p.), both vertebral arteries were electrocauterised to permanently occlude them. On the following day, both carotid arteries were occluded using aneurysm clips to induce global ischemia for 15 min. Then, the carotid atherosclerosis was removed to allow reperfusion. Rats that did not exhibit loss of consciousness, corneal reflex, pupillary light reflex, and righting reflex during ischemia were excluded from the experiment. The inclusion rate of each group ranged from 67 to 77 %, or 20 out of 26 mice to 20 out of 30 mice. The sham operation control group animals were subjected to the same surgical procedure but the carotid arteries were not occluded.

NaHS (Sigma-Aldrich 161527, USA) was dissolved in saline and given to rats at a concentration of 14 μM/kg via intraperitoneal injection immediately following the initiation of reperfusion. Control rats were intraperitoneally injected with saline.

### Grouping and temperature control

The rats were randomly divided into five groups (n = 20 each). Sham operation group (Sh), normothermic (36-37 °C) ischemia group (NT), mild hypothermic (32-33 °C) ischemia group (mHT), normothermic ischemia combined with NaHS group (NT + NaHS), and mild hypothermic ischemia combined with NaHS group (mHT + NaHS). Immediately following the initiation of reperfusion, mHT and mHT + NaHS rats were placed on an ice pack to cool down their rectal temperatures, which all reached 32 ~ 33 °C within 15 min. Rats were maintained at this temperature for 6 h by heat block. The rectal temperature of the other groups was maintained at 36 ~ 37 °C. After 6 h of reperfusion, rats were decapitated and hippocampus samples were immediately collected and flash frozen at -80 °C.

### Spectrophotometry

H_2_S content was measured on 4 samples from each group using the H_2_S content kit from Sigma-Aldrich (RAD171 USA) according to the manufacturer's instructions. Absorbance at 670 nm was measured using a spectrophotometer from SAPE (Shanghai, China). H_2_S content was then calculated based on a standard curve of H_2_S solution.

### Western blotting

To prepare samples for Western blotting, 4 samples from each group were cracked and homogenized on ice before centrifugation at 17,000 g for 60 min. Protein content of the lysates was then determined by Coomassie brilliant blue G250 binding assay and total protein content of the lysates was equalized prior to SDS-PAGE and transfer to nitrocellulose. Primary antibodies against NR2A, NR2B and p-CREB (1:1000; Cell Signaling Technology) were used for western blotting. Secondary antibodies were horseradish peroxidase (HRP)-labeled anti-rabbit or anti-mouse IgGs (1:5000, Sigma).

### Western blot quantification

Following ECL development, membranes were subjected to autoradiography and films were scanned and analyzed using an image analyser (Cheni Imager 5500, software V2.03). Western blot band intensity was quantified by measuring the mean pixel intensity of a fixed region of interest using Quantity One software. Background readings were taken from appropriate neighboring regions of the blot and subtracted from intensity measurements.

### qRT-PCR

BDNF mRNA levels were measured in 4 hippocampal samples from each group. Total hippocampal RNA was extracted using Trizol reagent according to the manufacturer’s instructions, and used for first strand cDNA synthesis using the TaKaRa kit. Target BDNF or control GAPDH sequences were then PCR amplified from cDNA. BDNF primer sequences were obtained from Shanghai Invitrogen ltd and were as follows; sense:5' CTGTTGGGGAGACGAGAT 3'; anti-sense:5' AGAAAGAGCAGAGGAGGC 3'; GAPDH primer sequences were sense: 5' AAGGTCGGAGTCAACGGATTT 3'; anti-sense: 5'AGATGATGACCCTTTTGGCTC 3'. The expression of BDNF relative to GAPDH was measured through qRT-PCR.

### Histology

Hematoxylin and eosin (HE) staining was used to examine pyramidal cells in the CA1 region. Immediately following reperfusion, 4 rats from each group were subject to thoracotomy through the ascending aorta cannulation. The rats were anesthetized and perfused with 200 ml of 4 % paraformaldehyde. Rats were decapitated and then incised after the optic chiasm 1 mm and 4 mm at the coronal. The middle section of the brain was harvested, fixed for 10 d in 4 % formaldehyde solution and paraffin embedded. The hippocampal dentate gyrus plane was cut into consecutive coronal slices 4 μm thick for HE staining of pyramidal cells.

### Statistical analysis

All data are expressed as mean ± standard deviation (± s.d.). Analysis between multiple groups was carried out by ANOVA using SPSS 13.0 software and any differences observed were further analyzed by least significant difference (LSD)-*t* test. p < 0.05 was considered to be statistically significant.

## Results and discussion

### Both NaHS and mild hypothermia result in increased hippocampal H_2_S levels

Since H_2_S is known to have a protective effect on brain function, we first wished to establish what effect administration of the H_2_S donor compound NaHS and mild hypothermia had on hippocampal H_2_S content. 100 rats were randomly divided into five groups: (1) Sh: sham operation group (2) NT: normothermic group (3) mHT: mild hypothermia group; (4) NT + NaHS: normothermic and NaHS treatment group (5) mHT + NaHS: mild hypothermia and NaHS treatment group. Transient (15 min) global cerebral ischemia was induced by the four-vessel occlusion method invented by Pulsinelli [13] and spectrophotometry was then used to measure the concentration of H_2_S in hippocampus.

In all instances, the hippocampal H_2_S concentration increased in test groups vs the sham operation group (p < 0.05). An additional elevation of H_2_S concentration was observed in NT + NaHS and mHT + NaHS groups relative to the NT group (p < 0.05), (Table 1). These results showed that global cerebal ischemia-reperfusion increased hippocampal H_2_S concentration. Furthermore, we confirmed that administrating the H_2_S donor compound NaHS effectively raised H_2_S levels in our experimental animals.

**Table 1:** H_2_S concentration in rat hippocampi as measured by spectrophotometer

Group	Levels of H_2_S (nmol/g)
Sh	15.2/g)0
NT	25.2 ± 2.0^*^
mHT	26.5 ± 3.5^*^
NT + NaHS	37.5aHS4^*, **^
mHT + NaHS	38.7SaHS^*, **^

*P < 0.05 compared to Sh; **P < 0.05 compared to NT (LSD-*t* test)

### H_2_S up-regulates NR2A and NR2B subunit expressions during brain recovery

While it is known that H_2_S induces NMDA receptor-mediated signaling following brain trauma, the underlying molecular mechanism is unclear. As the NR2A and NR2B receptors are broadly thought to have opposing effects on neuron survival following brain trauma [3], we postulated that their response to H_2_S would differ accordingly. Thus, we analyzed the relative protein levels of NR2A and NR2B following the different treatments described.

**Fig. 1:**
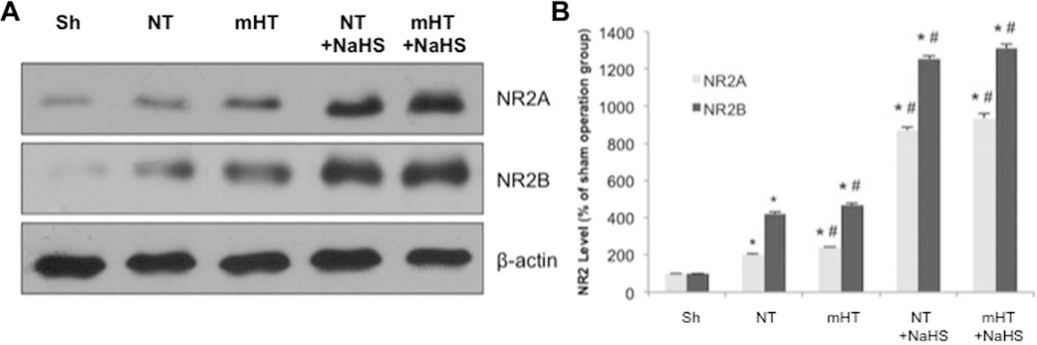
Both H_2_S treatment and mild hypothermia cause selective upregulation of NR2A and NR2B during brain recovery. **a**. Extracts from sham or treatment groups were immunoblotted with antibodies against NR2A or NR2B. β -actin was used as a loading control. **b**. Expression levels of NR2A and NR2B were quantified and plotted relative to those of sham operation group (Sh) for each protein. *, P < 0.05 compared to Sh, #, P < 0.05 compared to NT (LSD-*t* test)

Western blotting demonstrated that relative to the sham group, protein levels of both NR2A and NR2B increased for all animals subject to ischemia (Fig. 1a). These broadly followed the same pattern as seen with H_2_S levels. Global cerebral ischemia-reperfusion increased protein expressions of NR2A and NR2B. Treatment with NaHS caused an additional increase in the levels of both NR2A and NR2B, while mild hypothermia without NaHS treatment (mHT) had a small but significant elevation compared to NT (Fig. 1b).

The administration of ischemia-reperfusion alone (NT) or mild hypothermia following ischemia-reperfusion (mHT) resulted in a 2-fold increase in the level of NR2A and a 4-fold increase in the level of NR2B, and the addition of NaHS increased both levels by another 3 to 4-fold (Fig. 1b). Overall, our data did not support a hypothesized distinction between protein expression levels of NR2A and NR2B. Instead, both NR2A and NR2B are up-regulated in response to H_2_S and mild hypothermia.

### Up-regulation of NR2A and NR2B correlates with activation of the downstream pro-survival CREB signaling pathway

We next wished to investigate the downstream signaling pathway NR2A and NR2B up-regulation may mediate their pro-survival effect through. It has previously been reported that treatment of cultured rat neurons with NMDA resulted in increased phosphorylation of cAMP-response element-binding protein (CREB) [7]. To examine whether our treatments had similar effects, we analyzed the relative expression levels of p-CREB by western blot and its downstream pro-survival gene BDNF by qRT-PCR in the hippocampal tissue.

**Fig. 2:**
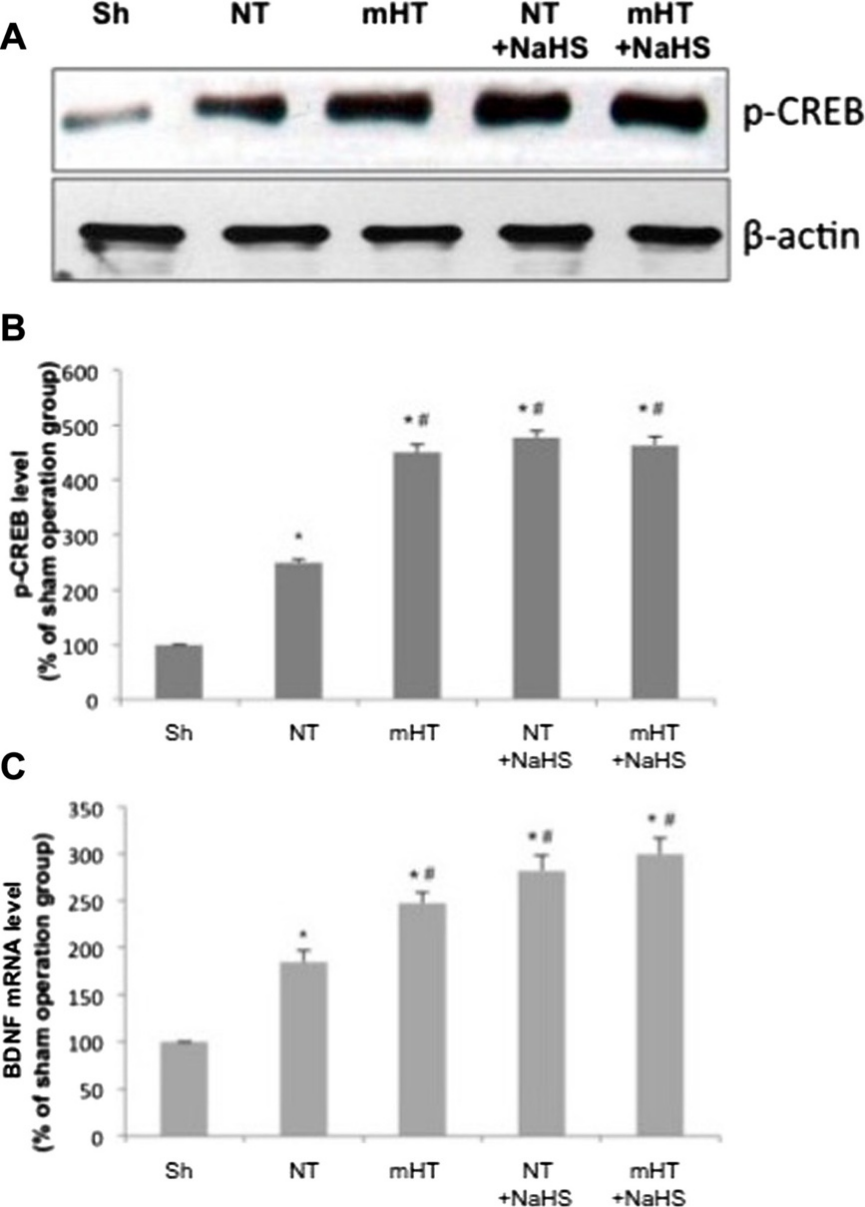
H_2_S up-regulation of NR2A activates the pro-survival CREB signaling pathway. **a**. Extracts from sham or treatment groups were immunoblotted with antibodies against phosphorylated-CREB (p-CREB). β-actin was used as a loading control. **b**. Expression levels of p-CREB were quantified and plotted relative to those of sham operation group (Sh) for each protein. *, P < 0.05 compared to Sh, #, P < 0.05 compared to NT (LSD-*t* test) **c**. qRT-PCR was used to assess levels of BDNF in total hippocampal RNA extracts from sham operation and treated groups. *, P < 0.05 compared to Sh, #, P < 0.05 compared to NT (LSD-*t* test)

In all treatment groups, there was a measurable increase in the levels of p-CREB and BDNF compared to those seen in the sham operation group (Sh) (p < 0.05). Additionally, groups treated with mild hypothermia, NaHS or both (mHT, NT + NaHS, mHT + NaHS, respectively) showed a significant increase in p-CREB and BNDF levels compared to the normothermic ischemia-reperfusion group (NT) (p < 0.05) (Fig. 2). The patterns of up-regulation of p-CREB protein levels and BDNF mRNA levels were similar between different treatment groups, consistent with a model where NaHS and mild hypothermia treatments induced the pro-survival CREB signaling pathway.

### Both NaHS and mild hypothermia treatments alleviate the ischemia-reperfusion damage in the CA1 region of hippocampus

**Fig. 3:**
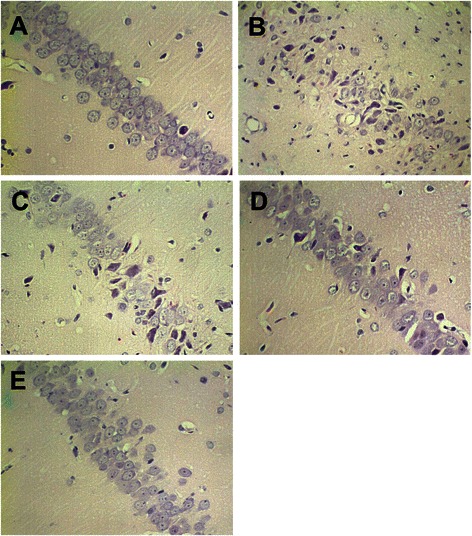
H_2_S and mild hypothermia protect rat hippocampal CA1 regions from ischemic injury. **a** – **e**. Immediately following reperfusion rat hippocampal regions were fixed and subjected to Hematoxylin-Eosin staining. Representative images from each treatment group are shown. **a**. Sham operation group (Sh) showed the typical organization of hippocampal CA1 region. **b**. Normothermic group (NT) demonstrated the damage caused by ischemia-reperfusion. **c**. Mild hypothermia group (mHT). **d**. NaHS group (NT + NaHS). **e**. Mild hypothermia + NaHS group (mHT + NaHS) showed that H_2_S and mild hypothermia reduced ischemia-related brain injury

Finally, we used hematoxylin and eosin staining (HE stain) to analyze the effect of the NaHS and mild hypothermia treatment on cell morphology and survival. We also calculated the number of irregularly shaped pyramidal cells per view for each group. In sham operation group (Sh), pyramidal cells possessed a uniform morphology in the rat hippocampal CA1 region. They packed together tightly to form 3-4 regular layers of cells. High magnification microscopy (400x) revealed that the nuclei of these neurons are large and round with 1 or 2 prominent nucleoli (Fig. 3a). We observed an average of 3.5 irregularly shaped cells per view (Table 2). Ischeamia-reperfusion induced a clear loss of this ordered cell morphology (NT). In NT, pyknosis occurred in the pyramidal cells, which lost their uniformed shape (Fig. 3b), and more than 50 irregularly shaped cells were present in each view (Table 2). Both mild hypothermia (mHT) and NaHS treatment (NT + NaHS) alone significantly prevented this degeneration, and less irregularly-shaped cells were observed (Table 2). In these treatment groups, there were higher counts of uniform cells with large round nuclei, which partially formed the ordered cell layers (Fig. 3c and Fig. 3d). When the rats were subjected to both mild hypothermia and NaHS (mHT + NaHS), the cell-damage phenotype was further rescued: fewer necrotic cells were visible and more cells regained their uniform shape and formed the ordered cell layers (Table 2, Fig. 3e). The histology results showed that both NaHS and mild hypothermia prevented cell damage induced by the ischemia-reperfusion insult. Furthermore, the protective effect of the two treatments are additive.

**Table 2:** Number of irregularly shaped pyramidal cells

Group	Number of cells per view (400x)
Sh	3.5 ± 1.1
NT	55.3 ± 6.3*
mHT	42.6 ± 5.7*^,^ **
NT + NaHS	36.7 ± 4.8*^,^ **
mHT + NaHS	23.3 ± 5.4*^,^ **

*P < 0.05 compared to Sh; **P < 0.05 compared to NT (LSD-*t* test)

## Discussion

H_2_S is a common signaling molecule generated endogenously by various tissues and modulates multiple neuronal function, including neural protection [14]. It regulates signaling kinases such as protein kinase A, receptor tyrosine kinases, and mitogen-activated protein (MAP) kinase. H_2_S also mediates the function of ion channels, in particular Ca^2+^ and K^+^ ion channels. Furthermore, it modulates the release of cuaminobutyric acid, N-methyl-D-aspartic acid, glutamic acid, catecholamine and other neurotransmitters [14]. NMDA receptors are important amino acid receptors linked to many signaling pathways in the CNS, mediating neuron survival, synaptic plasticity, as well as learning and memory. However, NMDAR-mediated excitotoxicity has been implicated in multiple neurodegenerative diseases. Importantly, several studies have shown that H_2_S can selectively modulate the functions of NMDARs [10].

Studies in mature cortical neuron cultures and rat models demonstrated that approximately 32.4 % of the synaptic spontaneous miniature excitatory postsynaptic currents (mEPSCs) are mediated by NR2B and 26.6 % of the extrasynaptic mEPSCs are mediated by NR2A [3]. Therefore, the differential expression of NR2A to NR2B can represent the extent of activation of NMDARs. We showed that the administration of ischemia-reperfusion alone (NT) or mild hypothermia following ischemia-reperfusion (mHT) resulted in a 2-fold increase in the protein levels of NR2A and a 4-fold increase in the protein levels of NR2B (Fig. 1b). It is possible that following global cerebral ischemia-reperfusion,excessive release of glutamates in extrasynaptic locations occurs, which stimulates NMDARs to over-express NR2B and cause neuronal cell death. This hypothesis would be consistent with the findings of Liu et al. [3]. However, in contrary to such previous studies, our data did not support a strong differential effect between NMDA subunits NR2A and NR2B. We showed that H_2_S and mild hypothermia up-regulated both NR2A and NR2B, and both treatments exerted protective effects. Thus, we cannot conclude that the NR2A exclusively exerts a protective effect and NR2B exerts a damaging effect. Overall, our findings align with other recent reports that refute the dichotomous separation between NR2A and NR2B [15, 16].

Previous studies have shown that membrane depolarization can activate NMDARs in the synapse, which then in turn mediate the expression and phosphorylation state of CREB, and promote survival of neuronal cells. CREB is a transcription factor that regulates the expression of a variety of genes. Specifically, phosphorylated CREB can activate the transcription of genes involved in cell survival [7]. In this study, the expression of p-CREB protein and transcription of BDNF mRNA increased after the administration of NaHS, suggesting H_2_S could activate the CREB signal pathway and promote expression of its downstream pro-survival gene, BDNF. H_2_S may up-regulates NMDARs and activates its downstream CREB signaling pathway, thus preventing brain injuries from ischemia-reperfusion.

While ischemia-reperfusion caused serious disruption to the organization of the hippocampal CA1 region, subsequent treatment with mild hypothermia and NaHS significantly prevented the pathological damage. Markarian et al. [17] showed that the mild hypothermia treatment must be deployed within 30 min after cerebral ischemia and maintained for at least 3 h or more to aid brain recovery. This is consistent with our finding of protective effect exerted by mild hypothermia that was deployed immediately after the injury and maintained for 6 h.

In this study, we also showed that mild hypothermia up-regulated expression levels of NR2A, NR2B, p-CREB proteins and BDNF mRNA. Thus, mild hypothermia may also up-regulate NMDARs and consequently activate the downstream pro-survival CREB signaling pathway. H_2_S concentration did not change significantly under mild hypothermia, implying that mild hypothermia may increase NMDAR expressions through a distinct pathway from H_2_S. Mild hypothermia has been suggested to influence brain recovery through a number of mechanisms, including inhibiting excessive release of neurotransmitters, such as acetylcholine, dopamine, norepinephrine, 5-serotonin and excitatory amino acids [18, 19]. The release of excitatory amino acids and glutamate could affect the expression of NMDARs, and mild hypothermia may function through such mechanisms to up-regulate expression of NMDARs and activate the CREB signaling pathway.

Overall, we showed that the administration of H_2_S or mild hypothermia could reduce ischemia-reperfusion injury, potentially through up-regulating NMDARs and activating the CREB signaling pathway. Additionally, we found that combining both treatments prevented neuronal cell damage to the greatest extent, and their additive effect may be exploited to effectively prevent reperfusion assaults in the hippocampus.

## Conclusion

Our results demonstrated that in the rat hippocampus, both exogenous administration of NaHS and mild hypothermia following global cerebral ischemia-reperfusion prevented damage to brain cells. Both treatments up-regulated NR2A and NR2B, as well as p-CREB protein expression levels and BDNF mRNA levels. Thus, H_2_S and mild hypothermia may exert neuro-protective effects through activating the CREB signaling pathway.

## Abbreviations

NMDA: N-methyl-D-aspartate
NR: N-methyl-D-aspartate receptor
CREB: cAMP-response element binding protein
PI3K: Phosphatidylinositide 3-kinases
Akt: Protein kinase B
NNOS: Nitric oxide synthase 1
NaHS: Sodium hydrosulphide
SD rats: Sprague-Dawley rats
SDS: Sodium dodecyl sulfate
PAGE: Polyacrylamide gel electrophoresis
HRP: Horseradish peroxidase
IgG: Immunoglobulin G
qRT-PCR: Quantitiative real-time polymerase chain reaction
BDNF: Brain-derived neurotrophic factor
GAPDH: Glyceraldehyde 3-phosphate dehydrogenase
CA1: Cornu ammonis 1
HE: Hematoxylin and eosin
s.d.: Standard deviation
ANOVA: Analysis of variance
LSD: Least significant difference
CNS: Central nervous system
MAP: Mitogen-activated protein
mEPSCs: Miniature excitatory postsynaptic currents
CRE: cAMP response element.
